# Introducing the trajectory Touchpoint technique: a systematic methodology for capturing the service experiences of palliative care patients and their families

**DOI:** 10.1186/s12904-020-00612-2

**Published:** 2020-07-10

**Authors:** Lynn Sudbury-Riley, Philippa Hunter-Jones, Ahmed Al-Abdin

**Affiliations:** University of Liverpool Management School, Chatham Street, Liverpool, L69 7ZH UK

**Keywords:** Patient experience, Narrative, Palliative care, Service research, Qualitative research, Person centred care, Patient journeys

## Abstract

**Background:**

Evaluation of palliative care services is crucial in order to ensure high quality care and to plan future services in light of growing demand. There is also an acknowledgement of the need to better understand patient experiences as part of the paradigm shift from paternalistic professional and passive patient to a more collaborative partnership. However, while clinical decision-making is well-developed, the science of the delivery of care is relatively novel for most clinicians. We therefore introduce the Trajectory Touchpoint Technique (TTT), a systematic methodology designed using service delivery models and theories, for capturing the voices of palliative care service users.

**Methods:**

We used design science research as our overarching methodology to build our Trajectory Touchpoint Technique. We also incorporated a range of kernel theories and service design models from the wider social sciences. We developed and tested our Trajectory Touchpoint Technique with palliative care patients and their families (*n* = 239) in collaboration with different hospices and hospital-based palliative care providers (*n* = 8).

**Results:**

The Trajectory Touchpoint Technique is user-friendly, enables systematic data collection and analysis, and incorporates all tangible and intangible dimensions of palliative care important to the service user. These dimensions often go beyond clinical care to encompass wider aspects that are important to the people who use the service. Our collaborating organisations have already begun to make changes to their service delivery based on our results.

**Conclusions:**

The Trajectory Touchpoint Technique overcomes several limitations of other palliative care evaluation methods, while being more comprehensive. The new technique incorporates physical, psychosocial, and spiritual aspects of palliative care, and is user-friendly for inpatients, outpatients, families, and the bereaved. The new technique has been tested with people who have a range of illnesses, in a variety of locations, among people with learning disabilities and low levels of literacy, and with children as well as adults. The Trajectory Touchpoint Technique has already uncovered many previously unrecognised opportunities for service improvement, demonstrating its ability to shape palliative care services to better meet the needs of patients and their families.

## Background

Ongoing evaluation of palliative care services is crucial. Good palliative care is a moral imperative [1] which has ‘social profit’ by positively impacting communities and families. Better understanding of palliative care helps future planning, which is vital due to the expected growth in demand. Demand drivers include medical technologies helping to increase longevity, but stagnant morbidity among an ageing population [2, 3] means non-communicable diseases now account for over two-thirds of global deaths [4], a trend set to continue as the post-war Baby Boom cohort ages [5]. Social trends including increased employment and smaller and more fragmented families means fewer familial carers [6], all of which drive demand for palliative care services. This situation is placing increasing financial pressures on palliative care providers to continually redesign their service offerings [7]. Consequently, assessing palliative care quality is a priority of many governments [8].

Palliative care terminology lacks consensus; sometimes it refers solely to the final stages of a terminal illness [4], while in other circumstances patients receive palliative care alongside life-extending treatments. The term hospice is equally confusing. US hospice is for people who have a prognosis of 6 months or less [9], while UK patients can access hospice care at any stage of life-limiting illness [10]. What is important is hospice is a philosophy of care that, like palliative care, focuses on care not cure [6]. In the study location (UK), over 200 hospice units exist, collectively supporting around 200,000 patients annually, free at the point of service. They receive an average of 32% of funding from the government, with the rest coming from fundraising [11]. Services are delivered through inpatient units, outpatient and day care centers, programs (e.g. Living Well, Bereavement), and clinics (e.g. Breathlessness Clinic), and hospice@home services, with the combination of services unit specific. UK Hospitals also offer palliative care; some have bespoke in-patient palliative care units, while others offer services to outpatients via a variety of clinics similar to those offered by hospices. Our study focuses on specialist palliative care services delivered either through hospice or hospital-based bespoke palliative care services.

Recently, palliative care providers have striven to expand and reach consensus regarding key quality of care indicators. In the UK, projects include ‘Listening Differently to Users’ [12] and ‘Every Moment Counts’ [13]. Well-known surveys include the Palliative Care Outcome Scales [14] aimed at patients, the SPARC questionnaire which is an early screening tool that ascertains palliative care needs [15], and the Carer Support Needs Assessment Tool [16] which aims to assess carer support needs. Examples of US initiatives include the Measuring What Matters program [17] and the CAHPS Hospice Survey [18]. There are also some commendable attempts to validate international measures, including the IMPACT project spanning five European countries [3]. Such initiatives focus on quality measurement and assessment techniques to drive service improvements [17].

However, major gaps in evaluation remain. Most of the available instruments comprise short satisfaction surveys. Though important, metrics fail to capture overall user experience and provide little information about how people felt. Some outcome measures are prone to floor and ceiling effects [19], and high misresponse levels [20]. Many measures are disease or location specific [9]. Systematic reviews reveal no survey that incorporates all areas important to palliative care patients and their families [21, 22] with many evaluations failing to take place within clinical practice [23] and often failing to detail systematic processes for developing quality indicators [24]. Currently, there is substantial variation in the quality of palliative care that people receive [1, 4, 25], and many recent calls for more research into ways of evaluating it [3].

Well-documented data capture difficulties when researching palliative care also hinder research. Difficulties include variable definitions of key issues [26], a hesitancy to discuss mortality [27], and a tendency for patients to formulate perceptions of palliative care solely on the basis of the interpersonal relationships they have with care providers which often leads to agreement that their care is superb [28]. Additionally, high participant attrition and a prevailing view that many hospice patients are too vulnerable to participate in research [8] has led to much research relying solely on retrospective views of family members [22], which of course misses key inputs from patients.

Capturing patient perspectives is pivotal for evaluating quality [29] and patient-centeredness. Despite considerable debate regarding what exactly comprises patient-centred care [30] and certainly the concept is not without its critics [31], patient-centeredness is part of the larger transition from paternalistic professional and passive patient to a more collaborative partnership, both in the clinical encounter and more recently to include organisational structures, cultures, leadership, and processes [32, 33]. People experience healthcare services holistically, so research should incorporate the whole range of service dimensions that may impact that experience. Taking the argument further, a shift in focus from ‘patient-centred’ to ‘person-centred’ care is not just about semantics. Person-centred care is “understanding what is important for the individual as a person, not just a patient with a condition. This understanding facilitates discussions and shared decisions about personalised care planning and management” [34]. Acknowledging that a universal definition of person-centred care is elusive, the Health Foundation [30] offers a person-centred care framework that 1) affords people dignity, compassion and respect; 2) is co-ordinated; 3) is personalised to fit around individual needs; and 4) is enabling in that that the traditional balance of power shifts from the professional being paternalistic to a more collaborative partnership. Additionally, person-centred care includes families and caregivers [31], which is important because family dynamics strongly influence individual experiences of palliative care [35]. Thus, being person-centred is about focusing care on the needs of the person rather than the needs of the service [36]. To shape the service around user needs, an in-depth understanding of these needs is required. This concept is at the heart of service design, which provides the techniques required for shaping services in a way that ensures they are person-centred, systematic, and collaborative [37].

It is on these foundations, i.e., the need to develop a novel way of understanding and shaping specialist palliative care services, using a methodology that is person-centred, that captures overall user experiences, irrespective of the nature of their illness and where they receive their care (hospital, hospice, at home), and that uncovers real opportunities for service improvement, that the Trajectory Touchpoint Technique (TTT) was designed. The aim of this paper is to introduce the TTT to palliative care research. First, we explain the underlying theories and concepts used to design the TTT. We then detail its application across multiple palliative care settings with a variety of palliative care service users. We define users as patients and their families. Finally, we evaluate the benefits of the new technique.

## Methods

To design our new TTT, we used design science research (DSR) as our overarching method. DSR is a process that focuses on creating innovative artefacts that solve organisational problems [38]. We followed the well-established DSR process [39], which incorporates three major phases: design, apply, evaluate. The design phase includes setting objectives and actual artefact design. The DSR process then moves to application, where the artefact in used real settings. These two steps – design and application – are detailed in our methods section here. The final DSR stage is evaluation. We evaluate our artefact – the TTT – against our objectives in the results section of the paper, and against alternative methodologies in our discussion.

### Solution objectives

Our preceding discussion outlined three major issues to consider when developing our objectives: the limitations of existing palliative care evaluation methods; specific data capture difficulties that beset palliative care research; and the need for a person-centred methodology able to capture the full service experience. Consequently, our objectives are that our new methodology should:

1. Be user friendly for all palliative care service users irrespective of illness type or place of care.
2. Systematically capture the lived experiences of service users, including subjective and abstract dimensions.
3. Enhance understanding of what ‘good palliative care’ means from the service user’s perspective.
4. Though innovative, be practical and functional from the researcher’s perspective.
5. Uncover feasible opportunities for improvement to palliative care services.

### Kernel theories

New artefacts start with kernel theories and current available methods and use these as building blocks to design better artefacts [39]. Evaluation of potential kernel theories from the service design and service systems literature led us to utilize six different concepts as our building blocks in designing the TTT, which we now explain.

#### Service blueprinting

Service blueprints entail breaking down services into their logical components, establishing different steps in service processes, and examining the execution of these steps in order to map a service at multiple levels [40]. Our service blueprint of ‘Blue Hospice’, our first collaborating organisation, comprised service user actions (e.g., accessing different information channels); service user-provider contact (with doctors, nurses, counsellors); support processes (e.g., admission processes) and the physical evidence that impacts the service experience. In sum, service blueprinting forces service designers to consider countless tangible and intangible elements that impact service experiences.

#### Servicescapes

Often overlooked in health, the physical, sensorial, and social dimensions of services are key issues, yet little research investigates the impact of servicescapes on the psychological and social needs of patients, as well as their coping resources [41, 42]. Servicescapes incorporate three major dimensions: ambient (e.g., cleanliness, olfaction, noise, furniture, visual attractiveness), design (e.g., architecture, comfort, signage), and social. Servicescapes have recently been recognized as extremely pertinent to people at end-of-life [43].

#### Touchpoints

Touchpoints represent any point of contact between a service user and any aspect of the service [44]. Touchpoints are also “clusters of experiential elements that foster product or service experiences” [45]. Hence our touchpoints incorporate issues central to palliative care, such as quality-of-life, spirituality, dignity, and ability to cope with grief; acknowledging that palliative care experiences comprise cognitive and emotional elements as well as physical, sensorial, symbolic, and social [42]. We wanted participants to discuss those touchpoints that they felt were important, hence a crucial part of the TTT is that people are free to select those touchpoints that resonate with them. Nevertheless we had to begin the process, so we identified as many potential touchpoints as possible, from the sources in Table 1.

**Table 1:** Touchpoint Identification

Source	Details	Benefits and Justification
Academic Sources	Academic Palliative Care Literature [e.g. [21–24]	Rich & deep understanding of different facets of palliative care, particularly useful for identifying neglected areas e.g., psychosocial, spiritual, and cultural aspects of care.
Academic Sources	Service Design Literature [e.g. [39, 46]	Ensured the methodology was patient centred.
Policy Documents	National and international policy documents [e.g. [4, 6, 9, 13]	Inclusion of physical, psychosocial and spiritual dimensions of palliative care
Hospice UK	Hospice audit tools [e.g. [17, 18]	Designed with input from a variety of stakeholders, these surveys were useful for insight into a wide range of palliative care issues.
Care Quality Commission (CQC)	CQC inspection reports [25]	Inspections provide insight into hospices legal requirements and regulations associated with Health and Social Care
Interviews with senior staff	Hospice Director; Clinical Director; Head of Fundraising.	Awareness of key strategic concerns.
Interviews: frontline & backroom staff	Nurses; Healthcare assistants; Receptionists; Volunteers.	Insight into hospice operations procedures.
Introspection	The authors shared their experiences of recent access to palliative care for relatives.	Helpful in revealing anticipations and reactions.
Unstructured interviews with 3 service users	No pre-planned questions, we simply listened to users narrate their recent palliative care experiences.	Unearthed the full linear journey, as well as revealing experiential service dimensions.
Observation	Close observation of different service dimensions on multiple occasions.	Ethnographic techniques and conversational analysis gave us rich insights into the practicalities and social dimensions of palliative care provision

#### Journey mapping

**Fig. 1:**
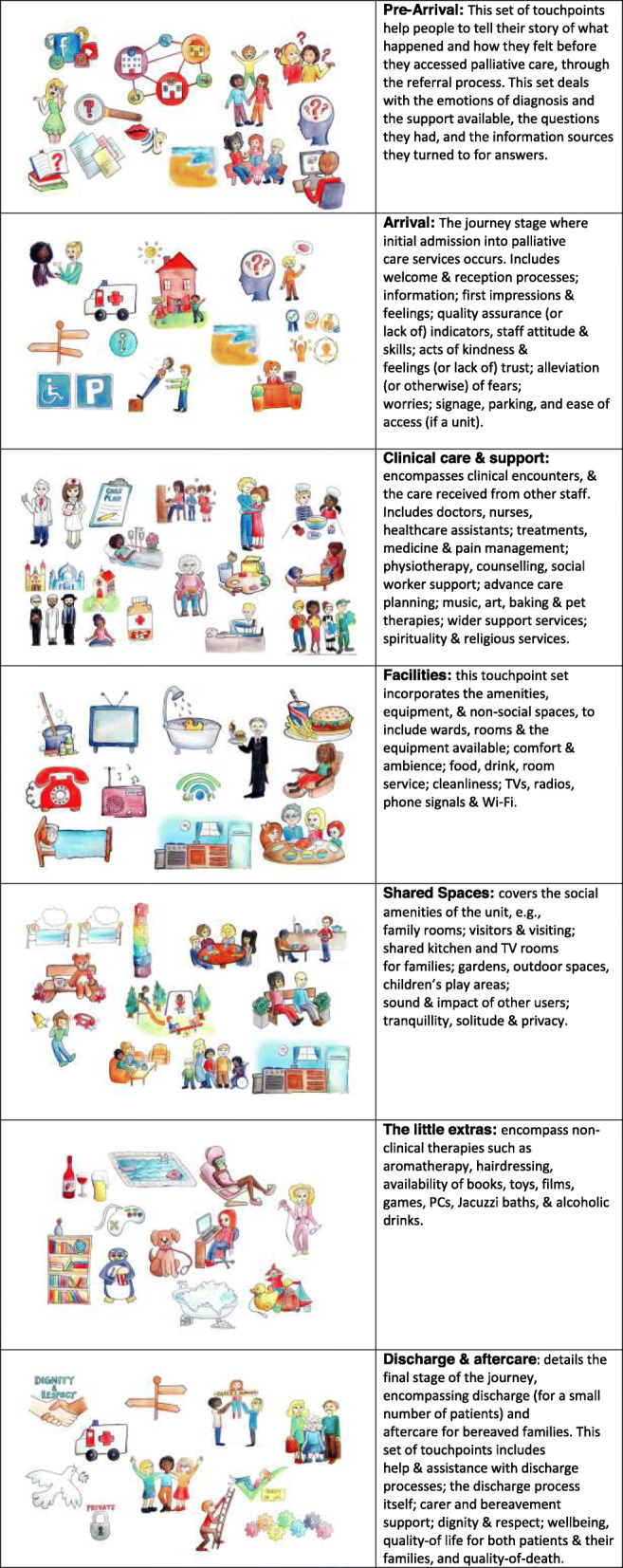
The 7 Sets of Touchpoints

The TTT examines the flow and processes involved in accessing different palliative care services. As such, it considers the uniqueness of individual journeys [10], which are often nonlinear and incorporate different locations (hospital, home, and hospice). This process, known as patient journey mapping, is reflected in the TTT together with emotional and physical touchpoints [42, 46]. Consequently, the TTT blends these two different approaches to incorporate opportunities to explore different experience dimensions. The starting point for a service journey is when the user needs the service, not when they first come into contact with the provider. In order to understand access to palliative care, our methodology needed to capture the point at which the patient needed palliative care, through to the present day. For some, the present day is days from death, for others it is a state of bereavement. Using the journey concept, we grouped the touchpoints into 7 categories, each representative of one stage or major aspect of the palliative care journey experience. The seven sets of touchpoints are detailed in Fig. 1.

#### Rich picture methodology

We used rich pictures in the form of cartoons to depict each potential touchpoint. Rich pictures enable people to explore their subconscious [47] and can act as an aide-memoir to help patients to construct their stories [48]. Increasingly used in health research, rich pictures are a systems thinking tool useful to explore complex phenomena [49], and can reveal issues missed when alternative methods are used [50]. In the first iteration of the TTT we used real photographs and cartoon drawings. However, during early testing of the TTT it became clear that people far preferred the cartoons, hence the images in Fig. 1. We printed each set of touchpoint images onto large laminated cards and uploaded them onto tablets. What is important is that respondents are free to use all, none, or some of the images.

#### Experienced based co-design (EBCD)

Designed for healthcare services in the UK, EBCD uses various qualitative techniques (interviews, observations, group discussions) to identify emotionally significant key elements – or touchpoints - of a healthcare service. A video then shares key findings with a group of staff and patients to explore ways to improve the care pathway. Central to EBCD is its qualitative focus on storytelling which allows for rich insights into experiences, ultimately enabling co-design and service innovation [51]. However, EBCD limitations include its inability to deal with multiple aspects of service, which leads only to small scale changes rather than systemic alterations to service delivery [52], yet it demands substantial resources [53]. Nevertheless, we took two key concepts of EBCD. First, narrative and storytelling is at the heart of EBCD [54] and we believed few other methods were capable of capturing the complexity and emotionality of palliative care services. Second, in recognition that patients and their families may experience healthcare services very differently to the intuition of staff [55], we designed a methodology to allow users to talk about touchpoints from their own perspective.

### Ethics

The full institutional ethical process took in excess of 6 months to complete and included University attendance and defence. The central research ethics considerations of anonymity, confidentiality, voluntary participation, informed consent, and vulnerability took on elevated scrutiny for this study. To address these considerations, all our communications (written and verbal) stressed both guarantees of anonymity and our independence from the palliative care providers. All communications stressed that participants were free to stop their narratives at any point, and without incurring any disadvantage. We allowed, and encouraged, patients to have a friend or family member with them when they narrated their stories; this was mandatory for children and optional for adult participants. Prior to accessing the children’s hospice, we returned to the Ethics committee and one of the amendments to our original approval was that we designed a child-friendly alternative information sheet (containing cartoon pictures) which was used by guardians to discuss the research with the child prior to attaining informed consent. A second amendment was that two researchers collected data when doing so at a patient or bereaved family’s home. We also designed a distress protocol which afforded participants the opportunity to contact the hospice counsellor, their family doctor, or their mental health provider. The protocol also included steps to enable us to refer to the hospice counsellor, with the participant’s permission. As a tight-knit research team, we had regular de-briefs and candid discussions between ourselves, and we too had access to counsellors if needed.

### Sample and participant selection

Blue Hospice comprised the first (and pilot) study, after which we collaborated with the range of palliative care providers shown in Table 2. Table 3 profiles the respondent samples. To ensure as much diversity as possible, we used purposive sampling [56] to select hospices and palliative care providers serving people of very different socioeconomic backgrounds (Table 2). We also ensured our sample comprised a full range of different service users, including inpatients, outpatients, family caregivers, and bereaved families. We incorporated different socio-economic statuses though, as Table 3 shows, it was relatively difficult to identify ethnic minorities, females were more willing to participate than males, and service users tended to be of higher socio-economic groups. The sample does have a wide age range, spanning 13 to 91 years old. In terms of sample size, the first three rounds of administration of the TTT (Blue, Red, Yellow hospices) suggested saturation occurs at around 20 narratives. As the hospital inpatient unit differed from the hospice, we oversampled but found saturation to occur at the same point. Because the Hospice@Home was a totally new service, the sample size is greater for this organisation.

**Table 2:** Collaborating Organisations

Alias	Geographic Area & Profile	Services
Blue Hospice	Elegant Spa towns and affluent, leafy suburbs in North of England. Significantly higher than national average of education levels and home ownership. Population is older than UK national average.	Inpatient Unit; Outpatient center; Caregiver & Bereavement Groups
Red Hospice	Prosperous urban area in South East England. Significantly higher than national average of education levels and home ownership. Population is older than UK national average.	Inpatient Unit; Day hospice; Hospice@home; Caregiver & Bereavement Groups
Yellow Hospice	Inner City in North West of England. City is classified as economically deprived with lower health status that national average, despite its younger average age.	Inpatient Unit; Day center services; Caregiver & Bereavement Groups
Green Hospice	Metropolitan District of North West England. Highly eclectic socioeconomic profile. This hospice serves areas that appear in the top and bottom 15% nationally most/least deprived areas. Population is aging.	Inpatient Unit; Day therapy unit; Hospice@home; Caregiver & Bereavement Groups
Purple Hospice	City in North East of England serving some rural areas too. Serves an area with significant pockets of social deprivation. Younger than average population.	Children’s Hospice with Inpatient Unit, Respite Services; End-of-Life Bereavement Suites; Sibling, parent & bereavement groups
Palliative Care Unit	City North West of England. Economically deprived city with large health and wealth inequalities. Age profile younger than national average.	Specialist hospital inpatient unit; referrals solely from the hospital; for most complex end-of-life needs
Specialist Palliative Care Services	Operating from a specialist NHS hospital based in a City in the North West of England, this specialist palliative care unit serves patients from the whole of the North West of England.	Multidisciplinary Specialist Palliative Care Team delivering a range of support & pain management
Hospice@Home	Market towns in affluent area of North England. Significantly higher than national average education, employment, and home ownership.	Palliative care delivered to the patient’s home during final expected 6 weeks of life

**Table 3:** Sample

	User Profile				***n***	Age							Gender		Ethnicity		SES		
	In-Patients	Out-Patients	Family Carers	Bereaved		Under 18	18−34	35−44	45−54	55− 64	65− 74	75+	M	F	WB	Other	AB	C	DE
**Blue Hospice**	4	14	12	8	38			1	7	4	15	11	10	28	37	1	17	17	4
**Red Hospice**	10	6	7	5	28			2		3	14	12	15	16	31	0	14	14	3
**Yellow Hospice**	4	14	4	9	31		1		6	4	5	12	12	16	27	1	12	12	4
**Green Hospice**	6	4	6	5	21		1		7	5	5	3	5	16	21	0	9	9	3
**Purple Hospice**	1	1	13	7	22	3	6	7	4	1	1	0	4	18	19	3	9	4	9
**Palliativ Care Unit**	9	N/A	16	4	29		2	4	4	8	5	6	13	16	26	3	17	10	2
**Specialist Palliative Care Services**	N/A	4	11	5	20		2	1	4	11	2	0	6	14	20	0	6	4	10
**Hospice @Home**	N/A	2	4	44	50		2	1	5	14	14	14	9	41	49	1	30	14	6
**Total n**	**34**	**45**	**73**	**87**	**239**	**3**	**14**	**16**	**37**	**50**	**61**	**58**	**74**	**165**	**230**	**9**	**114**	**84**	**41**

Recruitment technique depended upon the type of participant. For inpatients, during ward rounds, a doctor decided which patients were well enough to participate. A senior nurse then explained the study and gave those identified as well enough an information leaflet with details of how to volunteer. Recruitment of inpatients and relatives was via posters and information leaflets given out at reception. Outpatients and their families were given information sheets at clinics and caregiver groups. All recently (within 6 months) bereaved families and the small number of discharged patients were sent letters and in-depth information sheets containing contact details of the team if they had any further questions, and details of how to volunteer.

### Data collection

Most in-patients chose to narrate their experiences at their bedside, though others preferred to talk in a quiet room at the hospice/unit. Some bereaved families opted for a quiet room at the organisation, others elected for the researchers to visit them at home. All the hospice@home patients, their caregivers, and the majority of those bereaved requested home visits. Additionally, two researchers paired up to conduct the first five to six conversations for each organisation, ensuring a consistent style across each in-depth interview.

After an initial introduction, participants were reassured that the researchers were independent and all interviews were anonymous and in confidence. We clarified that rather than a traditional interview with lots of questions, the conversation was about their story. The touchpoint images merely act as examples and guides to enable people to narrate their experiences in a systematic manner. We made it clear that none of the touchpoint image sets were completely comprehensive: participants could talk around some or all of the pictures at each stage. Participants were also encouraged to talk about anything else in their journey that the touchpoints omitted. We stressed that the aim of the conversation was to capture their unique story of their palliative care journey.

Every interview began with the ‘pre-arrival card’, i.e., the beginning of the palliative care journey from the point at which palliative care was needed. For some, other sets of touchpoints were not needed, for example the final set ‘discharge and aftercare’ was not relevant for inpatients and their families, while the ‘facilities’ set was not relevant for hospice@home patients. The pictures enabled participants to focus on the details at each major stage of their journey, while they helped us to collect data in a systematic manner. The technique we employed allowed for a deep-dive in terms of probing experiences, issues, examples, and feelings, yet interviewer input was restricted mainly to questions such as, “and how did that make you feel?”

### Data analysis process

Manual thematic analysis [57] followed the verbatim transcription of the audio files. In step one, vastly aided by the touchpoint groupings, three experienced social science researchers separately examined the data within each set of touchpoints, identifying core and sub-themes. In step two the researchers shared their identified themes, reviewing and verifying the outcomes of step one. The TTT ensured systematic data collection, which had the added benefit of systematic and relative ease of data analysis. Between the researchers, extremely high levels of agreement arose around the identified themes.

## Results

Tables 2 and 3 detail the 8 different palliative care providers and the 239 participants who made the research possible. The multi-cite study spanned 4 years, during which time the TTT was designed, applied, and evaluated, with several refinements made based on extensive feedback. The resulting data set exceeds 2 million words, making any full discussion of the results beyond the scope of this paper, with its primary focus on introducing the new TTT and evaluation of how it overcomes several limitations of other palliative care evaluation techniques. Moreover, in design science research projects the results pertain to an evaluation of the artefact. Consequently, what we predominantly give attention to here is not so much the outcomes themselves but the benefits of the TTT as a methodology that enables a better understanding of all aspects of palliative care from the user’s perspective. We therefore structure this section around our five objectives.

Our first objective related to the methodology being user friendly with all palliative care service users irrespective of illness type or place of care. The cartoon images made people smile and ensured the researchers came across as friendly and informal. Unprompted remarks such as, *“It shows that you people appreciate me and you are listening”* (Patient), and *“oh what a clever way of doing it!”* (Patient) were often expressed. We used the TTT with one person who was illiterate, one with severe learning disabilities, with children, and with older people. The TTT did not exclude on the basis of illness: we used it easily with patients with cancer, motor neurone disease, severe COPD, and heart disease. Ease of use extended to participants feeling comfortable with the research instrument: “*it was thoughtful and you made me feel at ease”* (Bereaved). Others felt narrating their pathographies in this way was therapeutic, *“it was important for his story to be told and I’m glad I’ve done it”* (Bereaved). Thus, the first objective is met.

Certainly, the TTT achieved the second objective of systematically capturing the lived experiences of service users. The focus on journeys helped people to narrate their story in a methodical way, with many commenting on the depth of narratives, *“you have gone through everything so thoroughly, thank you very much”* (Patient). Unlike many palliative care data collection instruments, the TTT captures the whole experience rather than focusing solely on aspects of clinical care, and while the clinical care results are rich, detailed, and extensive, narratives also revealed a range of problems in the pre and post phase of palliative care provision. For example, there are often complications with referral, “*… the communication between the organisations is appalling … the left hand doesn’t know what the right is doing”* (Bereaved). People told of the grave difficulties in access, *“the situation at home was disintegrating”* (Bereaved), and too often people heard of services serendipitously, *“we were at the hospital and I spotted a leaflet – I don’t know why the GP had not mentioned it – we needed it a lot earlier … because we have struggled”* (Caregiver). Post core service issues also arose, with examples including lack of clarity among some inpatient’s relatives, “*we were really worried that we wouldn’t be able to cope with her at home”*, and outpatients, *“if I have a recurrence … will I be able to access some of the services? Hopefully I would, but erm …*” . The rich pictures proved to be excellent aide memoirs, with *“oh that reminds me!”* being common responses from both patients and their families. Hence the second objective, to systematically capture the lived experiences of service users, is met.

The TTT goes beyond the clinical encounter, and while our results revealed deep and vital issues around major dimensions such as pain management, ease of suffering, dignity, quality of death, etc., in line with objective 3 our results also revealed the significance of an assortment of seemingly small details that transpired to be very important. Particularly important for female cancer patients is haircare: “*her hair...It just got the point of being important”* (Bereaved). Aromatherapy, too, was an important aspect of wellbeing*, “It feels lovely … It was really relaxing … and I closed my eyes … It was marvelous” (Patient).* The therapeutic prominence of pets, and the range of salutary activates such as music, arts and crafts are also crucial service aspects*: “I keep going to the craft room and doing stuff, because it’s my way of escaping”* (Patient); *“I’ve been baking. I’ve never baked in my life …*. *And we’ve had such a laugh. I didn’t think I’d be here laughing” (Outpatient).*

Indeed, interaction with others who understand has a major impact on wellbeing: *“[My son] was with another little boy and they were chatting away and this little boy went ‘yeah, my sister’s dead now’ and my son said ‘oh really?’ he said ‘yeah, but she was a bloody nuisance anyway’ so you know, it was just, they can say whatever they want to say and it doesn’t matter, it doesn’t matter, they need to say it, they don’t mean it but they need to say it, so that’s why the hospice is brilliant” (Caregiver).* However, a preference for a shared ward over a private room is clearly dependent on the individual: *“he is a relatively quiet and private person, he would prefer a room of his own but that is not possible is it?”* (Caregiver), while for others the opposite is true, *“he went into a side ward he didn’t like it at all … he was very upset …*. *They moved him back eventually, but he was really really upset over that, maybe he should have been asked beforehand?”* (Bereaved). In line with our third objective, these examples clearly enhance our understanding of what ‘good palliative care’ means from the service user’s perspective.

Our fourth objective was to design a methodology that was innovative yet practical and functional for researchers to use. Collectively, this team of researchers has over 50 years’ experience in gathering and analyzing experiential data. In comparison to alternative data collection methods, we found the TTT to be extremely advantageous. First, the absence of an interview protocol allowed us to concentrate solely on the stories told by participants; enabling us to probe intensely to gather rich and deep data. People were astonished as to how much they had so say, having previously thought they could contribute little. Second, because the touchpoint sets follow a journey, the TTT enabled us to capture experiential data around different and sometimes complex service aspects in a highly logical way. Systematic data collection also made for systematic analysis, and striking was the relative ease of analysis and the high levels of agreement between us regarding emerging themes. Finally, the cartoon images somehow makes people feel more relaxed than they expected, which in turn makes it easier for researchers to initiate potentially distressing conversations. Indeed, expressions of gratitude at the sympathetic manner in which the TTT gathers data is summed up by comments such as: “*you’ve both been so kind, it was thoughtful and you made me feel at ease”* (Bereaved). Undoubtedly, the TTT achieves this objective.

That the TTT also met its final objective of uncovering feasible opportunities for improvement to palliative care services is succinctly articulated by this quote from a Hospital Trust executive, “*… in the development of this novel approach of patient engagement for evaluating the quality of clinical care, findings have illustrated the approach uncovers data which had hitherto remained hidden/undisclosed using traditional approaches. The collaboration seeks to maximize the learning and innovation from different clinical and academic sectors, to address a problem which has often been tackled from a singular perspective – with limited success …*. *We are already exploring additional areas where the TTT may provide novel solutions to longstanding clinical problems.”*

We discovered many examples of excellent care and outstanding staff. We fed back our findings and recommendations to each provider using a format whereby we couched our results in terms of the areas where palliative care was already excellent, and where it could be made better. Rather than detailed reports that many front-line staff members would have no access to, we fed back to staff with presentations based on the colourful touchpoint images. Around each journey stage, we explained those service areas (and there were many) that were already excellent. We then presented those areas where improvements could be made. We always used quotes from patients and their families when we presented these results. These quotes resonated: indeed, several times staff were visibly moved by the praise and admiration provided around those areas that were already excellent. This seemed to open up a willingness to push to do even better, and when areas where service did not reach excellence were presented in the words used by patients and families, staff became energized to rectify problems. We did not provide solutions: we merely provided the results and recommendations for change, synthesized into themes, and illustrated through quotes that resonated. We then summarised the major findings on large posters, each poster relating to one set of touchpoints. The posters were divided into 4 sections. One section illustrated (using quotes) those service areas that were already excellent; the second detailed those areas that could be improved. The third pertained to our recommendations for change. The fourth was blank: it merely invited staff to write (or use post it notes) their ideas for how change could be implemented from a practical perspective. Thus the start of the change process, via involvement, had begun. On the whole we found an appetite among staff to engage with the process and make an often very good service even better.

Our collaborating organisations have already begun to make changes based on the results, and a great deal of work is still ongoing for all our partners. Hence, for the sake of brevity, we limit our reporting of some of the changes to a brief overview, which are detailed in Table 4. As can be seen, there is no doubt that our final objective, to uncover feasible opportunities for improvement to palliative care services, has been exceeded; testimony to this is demonstrated by the many changes already implemented.

**Table 4:** Examples of Practical Changes Resulting from TTT

Area of change	Examples of resulting changes
External marketing communications	Design of new leaflets Extensive changes to ways information is provided on Websites New social media campaigns with patient stories New weekly local press articles
Increased public engagement	New public open events include ‘meet and eat’ and ‘book a hospice tour’, Significant increases in public engagement events such as fundraising activities, and participation in local fairs and fetes
New community liaison post	Works with community groups (e.g. Women’s Institute, church groups, school children) New volunteer recruitment drives to spread knowledge about hospice services and increase volunteer numbers.
Internal communications	New and updated bedside information folders Changes to staff name badges and lanyards for improved communication
Training	Staff training in non-cancer conditions (including dementia, heart failure, motor neurone disease, and advanced day therapies) The adoption of principles for situated learning with different teams Initiation of a new communication skills training programme which, to date, has been accessed by GP surgeries, hospitals, hospices, and community trusts in 9 different areas of the UK. Planned new patient communication training packages in conjunction with the education team at one of the Hospital Trusts
Improvements to patient support services	Changes to support patient communication and integration (including improved Wi Fi, and the purchase of plug-in bedside phones and new hearing loops, iPads, and talking boards) Increased counselling and initiation of several new support groups Improved access to spiritual support
New patient support services	Introduction of a bedside companion service Introduction of mobile hairdressers
Advance Care Planning	Feasibility project launched into Advance Care planning using an adapted version of the TTT rather than lists of questions
Service delivery methods	Changes to clinicians’ working rotas to improve continuity of care Changes to the servicescape (improved signposting, privacy measures, use of chapel, room layout), Improvements to equipment and facilities (chairs, sanitary bins, improved menus, audiobooks, headphones for managing noise).
Policy changes	Alterations to admission protocols to consider individual preferences for private rooms over shared wards Hospital policy changes surrounding the movement of palliative care patients
Improved coordination between providers	New training sessions delivered by a hospice for local GPs on a (regular) bi-annual cycle Monthly lunchtime training forum catering for relevant professionals (e.g., care home managers, ambulance staff) Creation of a new role of ‘community registered nurse’ for palliative care education and support for health and social care professionals Extension to a coordination of care programme within community nursing teams Creation of new community care coordinator role for better integration of hospice and other palliative care professionals

## Discussion

Recognising the many problems pertaining to palliative care assessment discussed in the beginning of this paper, Meier [58] recently acknowledged the difficulties of fashioning an empirical tool that is “feasible, actionable, and patient centred to directly assess the patients’ perceptions of hospice or palliative care” (p.352). The TTT is indeed an empirical tool designed with the patient and their families firmly at its centre. Interpretive methodologies, particularly narrative and stories, are by far the most powerful way of accessing personal human experience [54]. Not only does this overcome the limitations of surveys or even qualitative methods with pre-determined questions, it also takes a truly person-centred perspective. Person-centeredness epitomises the deepest parts of our humanity and represents the intangible components of patient-centred care [30]. The uniquely human and subjective nature of stories enable a deep dive into the lived experiences of palliative care service users, and not only confirmed and enriched understanding of known factors, but also brought new factors into view [59].

The TTT eliminates the need for predetermined questions and therefore avoids problems of metric-based measures which fail to reveal how people really feel. The TTT captures subjective and abstract dimensions of palliative care services that are important to users, effectively spotlighting aspects often omitted from surveys [21–23]. The TTT gives control to participants; they are free to talk about service aspects important to them, which may differ from those intuitive to providers [55]. Unlike surveys that are prone to misresponse and fall/ceiling effects [19, 20] the TTT encourages people to reveal their stories of their palliative care journeys deeply, extensively, and in great detail. Nor is the TTT disease or location specific. The TTT is inclusive: it overcomes the prevailing view that palliative care patients are too ill to participate in research [8] and allows extremely ill and vulnerable people to still have their voices heard. We utilised the TTT with cancer and non-cancer patients, with inpatients and outpatients, with families currently caring for people, and with bereaved families. No disease or location problems emerged in terms of administering the TTT. Unlike many alternative evaluation methods that fail to provide recommendations for improvement [21], the TTT uncovers feasible, actionable, and workable solutions to some palliative care problems.

The TTT also overcomes some well-documented data collection problems specific to palliative care. We found no evidence of the reluctance to speak about death and dying that is prevalent in society [27]. Rather, we found a real appetite among patients and their families to participate in the research. In fact, several times a patient who had not been invited to participate on the basis that they seemed too unwell, actually asked could they be considered after they had heard of the study from other patients. We also found the TTT enabled people to talk candidly even when they had formed an attachment to caring staff [28]. Perhaps it is the abstract nature of the cartoon pictures, which without doubt put people at ease, which enabled these frank discussions. Certainly prior research finds rich pictures are able to uncover previously hidden issues [48].

The design of the TTT enables data to be collected and analysed systematically, which is crucial given that literature often omits the systematic processes utilized to develop palliative care quality indicators [24]. Importantly, the TTT goes beyond the clinical encounter, viewing a person’s palliative care experiences as a holistic journey that incorporates myriad tangible and intangible touchpoints. This broader perspective is increasingly important in palliative care research [22, 32], but too often pre-designed healthcare evaluation surveys focus solely on the care provided to patients by healthcare staff, and fail to consider these wider issues [33]. Finally, the healthcare paradigm shift to person-centred care shifts our view of healthcare services from a dyadic exchange between clinician and patient to one that acknowledges that health services operate as complex networks of multiple entities. Our results demonstrate problems with key linkages in the current system and highlight some significant research gaps.

## Conclusions

Palliative care providers are already under pressure to conduct research that facilitates better understanding of user needs [12, 18, 22], not least because there is currently a struggle to meet the demands of adequate palliative care [4], and demand is set to rise dramatically [2]. Understanding palliative care user needs is important because person-centred care recognises that users have a right to inform decisions and collaborate with healthcare providers about services [34]. Palliative care assessment is also a priority for many governments [8], with quality assessment important for ascertaining both financial value [60] and social value, as well as the moral imperative to ensuring terminally ill people receive quality care [1]. Yet, relatively little research is conducted into this core element of healthcare [61]. Reviews of current palliative care evaluation tools have revealed major gaps in that they fail to assess all dimensions of care [21, 22] with many not tested in clinical practice [23]. Consequently there are many recent calls for more research into evaluating palliative care [13, 32]. In response we developed the trajectory touchpoint technique (TTT), a deep dive methodology that harnesses experiences for enriched understanding of palliative care and uncovers areas for improvement. The TTT incorporates a variety of social science perspectives as its kernel theories, including service blueprinting [40], recent thinking on service journeys [45], touchpoints [44], servicescapes [42], EBCD [51, 54], and rich picture methodology [47, 48]. The result is a unique methodology that captures the whole palliative care journey, including the linkages between the stages and phases of that journey. The methodology includes both patient and family experiences, which is important for palliative care services [9].

DSR demands that new artefacts are tested and validated in real situations [39]. We have demonstrated empirically that the TTT overcomes many limitations of existing methodologies. The TTT is not difficult to use, irrespective of user type or place of care. Its use of cartoons put people at ease and enable in-depth storytelling in a relaxing manner. The 7 groups of touchpoints aid systematic data collection across the whole palliative care journey. The technique incorporates all dimensions of the service that are important to the user; integrating the emotional, physical, sensorial, and social elements of the service. It also allows people to talk about areas specific to the final journey: issues such as spirituality, dignity, the need for counselling and support, and grief. Unlike the traditional methods of medical record review and retrospective data, data collection methods that reveal actionable results that lead to meaningful changes are crucial to implementing improvements to palliative care [24]. Our TTT uncovered numerous areas for palliative service delivery improvement, and our collaborating organisations have already begun to make improvements based on our results. Hence the TTT overcomes a limitation of many palliative care tools which often fail to make recommendations to address unmet needs [21]. The resource and time costs of the TTT are perhaps marginally higher that of a survey, but are a fraction of the $40,000 for a typical EBCD project [53], and yet provide far richer results. Limitations of the study include the fact that it has not been applied in all locations where palliative care occurs (for example, care homes) and is thus far limited to UK palliative care providers. As noted in our introduction, the UK differs from other countries so testing the TTT outside the UK is worthy of further research.

In research, storytelling [62], servicescapes [42], touchpoints [44], and rich pictures [49, 50] are not new. The ways these concepts, and others such as service blueprinting and patient journeys, are blended together makes the TTT unique. The TTT responds to calls for more comprehensive tools to assess palliative care, expanding the focus of inquiry from the clinical encounter to macro-level organisational systems [33] in order to enable discrete interventions that enhance patient-centred palliative care.

## Data Availability

The datasets used during the current study are available from the corresponding author on reasonable request.

## Abbreviations

DSR: Design Science Research
EBCD: Experienced Based Co-Design
NHS: National Heath Service
TTT: Trajectory Touchpoint Technique
